# Deep Learning in Precision Agriculture: Artificially Generated VNIR Images Segmentation for Early Postharvest Decay Prediction in Apples

**DOI:** 10.3390/e25070987

**Published:** 2023-06-28

**Authors:** Nikita Stasenko, Islomjon Shukhratov, Maxim Savinov, Dmitrii Shadrin, Andrey Somov

**Affiliations:** 1 Skolkovo Institute of Science and Technology, 121205 Moscow, Russiad.shadrin@skoltech.ru (D.S.); 2 Saint-Petersburg State University of Aerospace Instrumentation (SUAI), 190000 Saint-Petersburg, Russia; 3 Department of Information Technology and Data Science, Irkutsk National Research Technical University, 664074 Irkutsk, Russia

**Keywords:** GAN, CNN, precision agriculture, postharvest decay, fungi, image processing

## Abstract

Food quality control is an important task in the agricultural domain at the postharvest stage for avoiding food losses. The latest achievements in image processing with deep learning (DL) and computer vision (CV) approaches provide a number of effective tools based on the image colorization and image-to-image translation for plant quality control at the postharvest stage. In this article, we propose the approach based on Generative Adversarial Network (GAN) and Convolutional Neural Network (CNN) techniques to use synthesized and segmented VNIR imaging data for early postharvest decay and fungal zone predictions as well as the quality assessment of stored apples. The Pix2PixHD model achieved higher results in terms of VNIR images translation from RGB (SSIM = 0.972). Mask R-CNN model was selected as a CNN technique for VNIR images segmentation and achieved 58.861 for postharvest decay zones, 40.968 for fungal zones and 94.800 for both the decayed and fungal zones detection and prediction in stored apples in terms of F1-score metric. In order to verify the effectiveness of this approach, a unique paired dataset containing 1305 RGB and VNIR images of apples of four varieties was obtained. It is further utilized for a GAN model selection. Additionally, we acquired 1029 VNIR images of apples for training and testing a CNN model. We conducted validation on an embedded system equipped with a graphical processing unit. Using Pix2PixHD, 100 VNIR images from RGB images were generated at a rate of 17 frames per second (FPS). Subsequently, these images were segmented using Mask R-CNN at a rate of 0.42 FPS. The achieved results are promising for enhancing the food study and control during the postharvest stage.

## 1. Introduction

According to the data provided by United Nations, the human population has grown to 8 billion people [1], and it is expected to increase up to 9.8 billion by 2050 [2]. The growing population will need more sustainable and affordable food sources. It increases the importance of agriculture in the light of sustainable development. In terms of food producing and quality control, agricultural challenges can be divided into preharvesting, harvesting and postharvesting stages [3]. Each stage includes various factors that should be taken into account in order to minimize food losses. During the postharvest stage, farmers primarily concentrate on factors that impact the shelf-life of harvested products during storage and transportation. These factors include temperature [4], humidity [5], as well as the use of gases and chemicals in food containers [6,7]. Each crop has its own number of factors affecting the shelf-life during the postharvest stage, and these factors should be also taken into account [8]. Disparagement of one of these factors or violation during the storage or transportation may result in postharvest losses of food products. Examples of postharvest losses in stored fruits and vegetables include decayed and spoiled areas, often attributed to mishandling, hygiene issues, inadequate humidity control, improper temperature management, and mechanical damages [9]. These factors contribute to the deterioration and loss of quality of stored subjects.

Apple is one of the most popular harvested and cultivated crops. Its global production achieved 93 millions tonnes in 2021 [10]. It is one of the major reasons to monitor apple fruits quality during all the above-mentioned stages to prevent postharvest losses and to avoid potential economic losses. However, there are special factors affecting apple quality during the postharvest stage, e.g., water as loss in apple fruits [11], residual pesticides [12], or concentration of carbon dioxide, ethylene, ethanol or ammonia surrounding apples due to insufficient ventilation in the storage facility [13]. The most common non-destructive methods for preventing postharvest losses include the control of objects using RGB video cameras and sensors [14], near infrared (NIR) data [15], gas sensing spectroscopy [13], fluorescence spectroscopy [16], magnetic resonance imaging (MRI) [17], and even electronic nose [18]. Nevertheless, postharvest losses are still estimated in the range of 40–50% [9]. It should be noted that the control of apple fruits at the postharvest stage is quite comprehensive, making it difficult to monitor each fruit at each step, while any damage may lead to a fungi infection [19] in the stored fruits and also to the formation (and even a rapid growth) of rotten areas which are also known as decayed areas [20]. Moreover, these areas are not well seen visually at early stages, and the decay growth process can be quite dynamic [21].

Artificial intelligence (AI) and its domains, including machine learning (ML) and deep learning (DL), in conjunction with the latest achievements in computer vision (CV), remote sensing, wireless sensing technologies, and Internet of Things (IoT), have provided the added value in a number of application including the space domain [22], medicine [23], power engineering [24], agriculture [25] and food supply [26]. For example, farmers rely on CV for crop quality management, e.g., plant growth monitoring [27], fruit detection [28], disease detection [29] and weed detection [30]. It is necessary for improving the food quality of each plant at preharvest, harvest, and postharvest stages, respectively. Also, there is a set of CV-based approaches for postharvest losses estimation and the evaluation in stored crops [31,32,33]. However, some postharvest losses, e.g., fungi or postharvest decay zones, should be detected immediately, since the visible decayed or fungi zones (acquired visually or with RGB cameras and sensors) in stored plants may indicate their serious spoilage if we use other types of imaging data, e.g., NIR or thermal imaging, to monitor their quality. This monitoring process requires a special device and equipment, e.g., multispectral or hyperspectral cameras, which are expensive and often not easy to use, given fast detection of defects is still extremely challenging.

In this article, we present an approach based on the application of generative adversarial network (GAN) and convolutional neural network (CNN) for early detection and segmentation of decayed and fungi areas in stored apples at the postharvest stage using visible near-infrared (vis-NIR, or just VNIR) imaging data. We show how artificially generated VNIR imaging data can be used for early postharvest decay detection in stored apples and examine whether GAN- and CNN-based approaches can achieve promising results for image segmentation tasks. The idea of the proposed approach can be divided into two parts:

- Generation of VNIR imaging data containing the stored apples with postharvest decay and fungi zones using the GAN technique.
- Segmentation of generated VNIR images using the CNN technique in order to detect the decayed and fungi zones in the stored apples.

In this research, we study the original and generated VNIR images containing apples of four varieties with several treatments in order to simulate various occasions with apples during the storage. The aim is to present an approach based on the DL techniques combining the GAN and CNN models, for instance, with segmentation of postharvest decay zones and fungi areas. The GAN model will provide the procedure of NIR images synthesis from the input RGB data, while the CNN model is supposed to be used for the instance segmentation of generated images. This is important for the proposed approach, as we aim to train and validate our models to detect the postharvest decay zones and fungi areas separately from each other. For realizing this idea into practice, we propose the following stages.

First, we need to select a GAN based model for the NIR images generation from the input RGB data. There are many available networks, but for the image-to-image translation tasks the following architectures Pix2Pix [34], CycleGAN [35], and Pix2PixHD [36] are mostly applied in agricultural domain [37,38,39,40,41,42,43]. We compare Pix2Pix, CycleGAN, and Pix2PixHD models using the dataset containing the paired RGB and NIR images. We are going to work with the images acquired in VNIR range since it includes the full visible spectrum with an abutting portion of the infrared spectrum [44]. The paired images collected in the visible (380–700 nm) and VNIR (400–1100 nm) ranges are required to make sure that the decayed and fungal traits in stored apples are the same for these two ranges. Section 3.1.1, Section 3.1.2, and Section 3.1.3 provide detailed information about the Pix2Pix, CycleGAN, and Pix2PixHD models, respectively.

Second, it is necessary to choose the CNN model for the decayed and fungal areas segmentation in the synthesized VNIR images. In this work, we implement a Mask R-CNN model due to the Feature Pyramid Network (FPN) and ResNet101 backbone, which allow for generating the bounding boxes (object detection) and segmentation masks (instance segmentation). In [45], we have compared the Mask R-CNN to such applied CNN-based models as U-Net [46] and Deeplab [47] for early postharvest decay detection, and Mask R-CNN achieved the highest performance in terms of average precision, namely 67.1% against 59.7% and 56.5%, respectively. Moreover, the Mask R-CNN model generates the bounding boxes and segmentation masks of the postharvest decay and fungal zones separately from each other. This is a so-called ‘a tried and tested’ method, and that is why we use Mask R-CNN as a CNN-based segmentation model. We discuss the Mask R-CNN model in more detail in Section 3.1.4.

**Figure 1:**
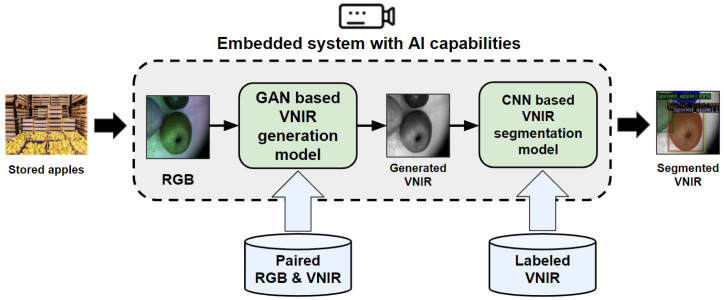
Diagram summarizing the proposed approach for the application of segmented VNIR imagery data via deep learning for early postharvest decay prediction in apples.

Finally, our plan is to implement the proposed approach and execute it on a Single Board Computer (SBC) with the AI capabilities. This implementation will serve as an evaluation platform for generating segmented VNIR images that highlight any postharvest decay and fungal zones on apples. These zones may be imperceptible to the human eye, but can be detected and selected through our system. We use NVIDIA Jetson Nano as an embedded system with AI capabilities for evaluation. It is a compact and powerful SBC supplied with the accelerated libraries for computer vision and deep learning applications, and is widely used for different real-time problems in agriculture including weed control [48], soil mapping in greenhouse [49], and harvest product detection [50,51,52,53,54]. That is why the presented research is supposed to be an alternative solution for the high-cost NIR hyperspectral devices used for the early postharvest decay detection and prediction for stored food. Figure 1 illustrates the proposed approach.

The contribution of this work is as follows:

- Two experimental testbeds for paired RGB and VNIR imaging data collection under various environmental (temperature and humidity) conditions.
- Application of CNN models, for instance, on the segmentation of decayed and fungi areas in apples at the postharvest stage.
- Separate segmentation of fungi zones and postharvest decay areas in stored apples using the CNN model.
- Application of the trained CNN-based model for the instance segmentation of postharvest decay zones and fungi areas in VNIR images generated by the GAN-based model.
- Implementation of the proposed approach based on the GAN and CNN techniques for postharvest decay detection, segmentation and prediction using generated VNIR imaging data on a low-cost embedded system with the AI capabilities.

This article is organized as follows: Section 2 provides an introduction to relevant research works aimed at early postharvest decay detection and prediction in apples using RGB and VNIR imaging data with the CV and ML methods. Section 3 presents the methods used in this work. Section 3.3 demonstrates the experimental testbeds used for RGB and VNIR imaging data collection and describes the procedure of data annotation. Section 4 shows the results of the comparison of the GAN techniques applied to VNIR images generation from the RGB ones (see Section 4.1). It also presents the application of the CNN technique, for instance, on the segmentation on the generated VNIR images (see Section 4.2), and describes the embedded system running the proposed GAN and CNN (see Section 4.3). Conclusions and discussion of the future work are summarized in Section 5.

## 2. Related Works

### 2.1. CV Approaches Based on CNN Models Using RGB Imaging Data

CV techniques with the implementation of ML and DL methods are becoming one of the most useful tools for fruit quality estimation and evaluation at the postharvest stage.

The majority of approaches are based on the collection and analysis of visible morphological traits, such as changes in fruit shape, size, or color during the storage, from stored fruits with CNN models using RGB images as the most acceptable and user-friendly type of data. RGB imagery is closely similar to human vision because red, green and blue are the primary colors in these color models, which makes the process of visible non-destructive quality monitoring and defect detection of stored food production easy and understandable [55]. The majority of cameras and devices for RGB imaging data collection contain a patterned Bayer filter mosaic consisting of squares of four pixels with one red, one blue and two green filters [56]. Usually, the Bayer filter is located on the camera chip.

Generally, a CNN model contains convolutional and pooling layers (added one by one), flatten, fully connected layer and softmax classifier. The convolutional and pooling layers are used in the features extraction part, while the classification part involves the flatten, fully connected layers and softmax classifier. When the image reaches the input layer, a filter in the convolution layer allows it for the selection of feature neurons. An activation function (Sigmoid, Rectified Linear Unit (ReLU), or Softplus) is added to obtain nonlinear results by passing feature neurons through it, and the resulting feature map size is reduced by the pooling layer functions. The flatten layer is the first input layer for the classifier model as it keeps the feature map from the convolution layers. The fully connected layer transforms the obtained feature neurons into a matrix, which performs the classification function with a classification method.

In this way, the CNN structure showed its efficiency in classification, and then in detection and segmentation tasks using RGB imaging data. For example, the automated banana grading system was reported in [57] where a fine-tuned VGG-16 Deep CNN model was applied for banana classification using such traits as skin quality, size, and maturity with the acquired RGB imagery data. A similar approach was proposed in [58] where the VGG-16 model was trained to predict the date of the fruit ripening stage using RGB images with an overall classification of 96.98%.

In [59], the authors developed an automated online carrot grading system, where a lightweight carrot defect detection network (CDDNet) based on ShuffleNet [60] and transfer learning was implemented for carrot quality inspection using RGB and grayscale images. The CDDNet was compared to other CNN models including AlexNet, ResNet50, MobileNet v2, and ShuffleNet, and it demonstrated good performance in terms of detection accuracy and time consuming for binary classification of normal and defective carrots (99.82%), and for classification of normal, bad spots, abnormal, and fibrous root carrots (93.01%). However, the images of carrots contained the carrots of different size and appearance, and the idea of the presented approach was to detect the carrots with visible defects without taking into account the spoilage stage of the defective carrots. Moreover, there was no mention of a possible situation when the carrots are infected, but still there are no visible traits of spoilage.

In [61], the authors report on the implementation of the DeeplabV3+ model [62] with a classical image processing algorithm, e.g., threshold binary segmentation, morphological processing and mask extraction for banana bunches segmentation during sterile bud removal (SBD) on the total of 1500 RGB images. Moreover, YOLOv5-Banana model [63] for the banana fingers segmentation and centroid points extraction, while edge detection and centroid extraction of banana fingers included binarization, morphological opening operation, canny edge detection, and extracting centroid point set. DeeplabV3 was reported to achieve a detection accuracy rate of 86%, mean intersection over union (MIoU) of 0.878 during the debudding period for target segmentation, and the mean pixel precision of 0.936. YOLOv5-Banana achieved 76% detection accuracy rates for the banana bunches during the harvest period. The authors also designed and presented the software to estimate the banana fruit weight during the harvest period.

In [64], several CNN-based models including VGG-16, VGG-19, ResNet50, ResNet101, and ResNet152 were compared to each other for such physiological disorders classification in stored apples as bitter pit, shriveling, and superficial scald. The authors acquired a dataset containing 1080 RGB images (dataset-1) of apples and 4320 augmented images (dataset-2) with the aim to improve data representation during model training and to consider apple position under the monitoring camera and lighting conditions during the storage. The CNN-based models were used and compared for feature extraction, while such classical ML methods as support vector machines (SVM), random forest (RF), k-nearest neighbors algorithm (kNN), and XGBoost were used for the extracted features classification. The highest average accuracy was reported for the VGG-19 model in conjunction with the SVM method in the dataset-1 and dataset-2 with 96.11 and 96.09%.

### 2.2. Machine Learning and Deep Learning Methods for NIR Data Analysis

NIR spectroscopy covers spectral regions from 780 to 2500 nm that cannot be seen with human eyes, but it allows for obtaining spectral information from ten (generally, referred to as multispectral data [65]) and to more than a hundred wavebands (referred to as hyperspectral data [65]). Measurements performed in the visible (380–700 nm), visible near-infrared (vis-NIR, or just VNIR, 400–1100 nm), and NIR (780–2500 nm) ranges provide the user with more detailed information on the chemical composition of scanned samples. In our case, by samples we mean stored plants, crops and fruits. The state-of-the-art cameras and devices for the hyperspectral data acquisition provide not only spectral information about the scanned samples, but also allow the users to obtain the images of scanned zones in the range of device bands. Spectral information on chemical composition from a wide range of wavebands has simplified the procedure of food quality monitoring and defect detection at the postharvest stage. Moreover, not only the decay zones may occur in stored fruits, but also some fungi like *Sclerotinia sclerotiorum* [66], *Penicillium expansum* [67], *Botrytis cinerea* [68], *Botryosphaeria dothidea* [69] and many others, which should be immediately detected at the early stage. Otherwise, the appearance and growth of decayed and fungi zones may lead to the loss of all stored fruits. It is vital to distinguish various types of postharvest losses, e.g., postharvest decay, and diseases, e.g., various fungi varieties, since each type of loss requires a special type of treatment or removal of spoiled samples from the storage. It should be noted here that the formation of fungal areas may not always lead to the formation of decayed areas. That is why we should detect and identify the fungi and postharvest decay zones separately from each other [70,71,72].

Both classical ML methods and the DL techniques based on the CNN models are widely used for postharvest losses evaluation in stored plants using VNIR and NIR imaging and spectral data.

In [73], the authors compared several ML methods including linear discriminant analysis (LDA), random forest (RF), support vector machines (SVM), kNN, gradient tree boosting (GTB), and partial least squares-discriminant analysis (PLS-DA) for early *Codling Moth* zones detection in “Gala”, “Granny Smith”, and “Fuji” stored apples. The research was carried out at the pixel level using NIR hyperspectral reflectance imaging data in the range of 900–1700 nm with an optimal selection of wavelengths. GTB was reported to obtain better results at a pixel level classification with 97.4% of total accuracy for validation dataset.

In [74], the authors implemented the AlexNet model for detecting pesticide residues in postharvest apples using hyperspectral imaging data. There were 12,288 hyperspectral acquired images for the training set and 6144 images for the test set in the 865.11–1711.71 nm range (the camera included 256 bands) and with 3.32 nm spectral resolution. Otsu segmentation algorithm [75] was used for the apples and pesticide residue positioning (they were the regions of interests, or just ROIs), while deep AlexNet [76] provided pesticide category detection. AlexNet was reported to show better results in terms of detection accuracy and time consumption in comparison to the SVM and kNN algorithms (99.09% and 0.0846 s against 74.34% and 11.2301 s, and 43.75% and 0.7645 s, respectively).

As we can see, NIR hyperspectral and multispectral imaging data ensures early disease detection with more details than RGB imaging, but also requires sophisticated equipment, which usually includes a camera with wavebands, imaging spectrograph (or spectrometer), sample stage, illumination lamps and lightning system, as well as supplementary software and devices for processing and capturing NIR data and images [77,78,79]. However, this is the reason why hyperspectral imaging devices are so expensive and may cost from thousands to ten thousand USD [80]. These high prices reduce the availability and usage of hyperspectral cameras for farmers and food selling companies to perform food quality control at postharvest stages. This issue has raised a demand for developing new approaches for NIR imaging data generation without using high cost hyperspectral systems.

### 2.3. GAN-Based Models for RGB and NIR Data Analysis

Generative Adversarial Networks (GANs) and, in particular, conditional GAN (cGAN) [81] have demonstrated their effectiveness in a variety of tasks in the agricultural domain including remote sensing [82], image augmentation [83], animal farming [84], and plant phenotyping [85]. The general idea of GAN is based on the usage of two neural network models, where the first network is called generator (generative part, G) and its goal is to create plausible samples, while the second network is called discriminator (adversarial part, D), and it learns to verify whether the created plausible sample is real or fake. GANs are also applied for the so-called image-to-image translation tasks, i.e., where there is a need for high-quality image synthesis from one domain to another. For example, GAN-based models were successfully applied for the multi-channel attention selection in the RGB imagery considering an external semantic guidance in [86,87], MRI data estimation in [88], diffusion models evaluation [89], and NIR imaging generation from the input RGB images in [82,90,91].

Therefore, the approaches based on GAN models allow synthesizing high-quality NIR images from the input RGB images while saving detailed spectral information. At the same time, it is crucial not only to transform the image together with all the relevant information, but also to segment various types of postharvest diseases and defects separately from each other in stored food production in order to choose the specific processing strategy for defected or spoiled food samples. At present, most GAN models provide only the images transformation from one domain to another, but not object detection or instance segmentation operations in the synthesized images. However, as shown in Section 2.1, CNN models demonstrate reasonably good results for the object detection and instance segmentation both for the RGB and the NIR images.

## 3. Materials and Methods

### 3.1. DL Techniques

#### 3.1.1. Pix2Pix

The Pix2Pix model [34] is a type of cGAN that has been demonstrated on a range of image-to-image translation tasks, such as converting a satellite image to corresponding maps, or black and white photos to color images. In conditional GANs, the generation of the output image is conditional on the input image. In the case of the Pix2Pix model, the generation process is conditional on the source image. The discriminator covers both the observed source image (*domain A*) and the target image (*domain B*) and must determine whether the target is a plausible transformation of the source image. The generator is trained via the adversarial loss which encourages the generator to make plausible images in the target domain. The generator is also updated via $L_{1}$ loss measured between the generated image and the expected output image. This additional loss encourages the generator model to create the plausible translations of the source image. Mathematically, the whole process in Pix2Pix can be defined as:

(1) $$L_{cGAN}\left(G,D\right)=E_{x,y∼p_{data(x,y)}}\left[logD\left(x,y\right)\right]+E_{x,z∼p_{data(x,z)}}[log\left(1-D\left(x,G\left(x,z\right)\right)\right]$$

where *G* is the generator, *D* is the discriminator, *x* is the observed image, *y* is the target image, *z* is the random noise vector, and λ controls the relative importance of the two objectives between *domain A* and *domain B*. The following objective function is used to train the model:

(2) $$G=arg\min_{G}\max_{D}L_{cGAN}\left(G,D\right)+\lambda L_{L1}\left(G\right)$$

**Figure 2:**
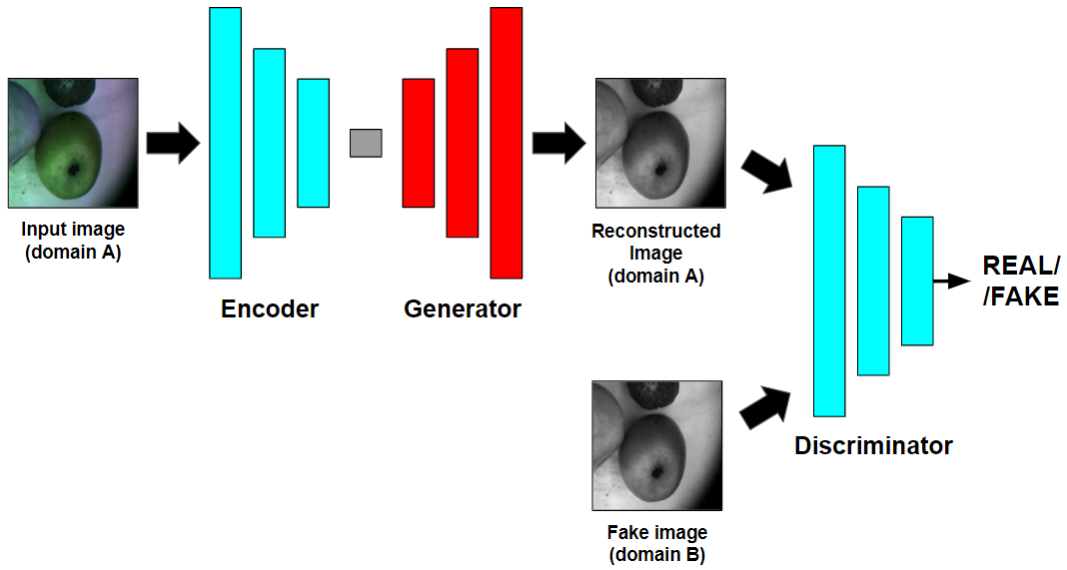
Pix2Pix block diagram.

Pix2Pix requires perfectly aligned paired images for the training procedure. In this research, the CNN-based architecture is used both as the generator and the discriminator. Generally, the U-Net model [46] is applied in Pix2Pix as a generator. U-Net trains to generate the images from the images in *domain A* similar to the images in *domain B*. The discriminator is usually a PatchGAN (which is also known as Markovian discriminator [92]), and it trains simultaneously to distinguish the generated images from the real images in *domain B*. The reconstruction loss measures the similarity between the real images and the generated images. Figure 2 shows the block diagram of Pix2Pix.

#### 3.1.2. CycleGAN

The goal of the CycleGAN model [35] is to learn the mapping G:X→Y such that the distribution of images from G(X) is indistinguishable from the distribution *Y* using an unpaired set of image pairs. This mapping is coupled with an inverse mapping F:Y→X and a cycle consistency loss introduced to enforce F(G(X))≈X and vice versa due to the reason that it is highly underconstrained. For the mapping function G:X→Y and its discriminator $D_{Y}$

(3) $$L_{GAN}\left(G,D_{Y},X,Y\right)=E_{y}\left[logD_{Y}\left(y\right)\right]+E_{x}[log(1-D_{Y}\left(G\left(x\right)\right]$$

and the objective is as follows:

(4) $$G,F=arg\min_{G,F}\max_{D_{X},D_{Y}}L\left(G,F,D_{X},D_{Y}\right)$$

**Figure 3:**
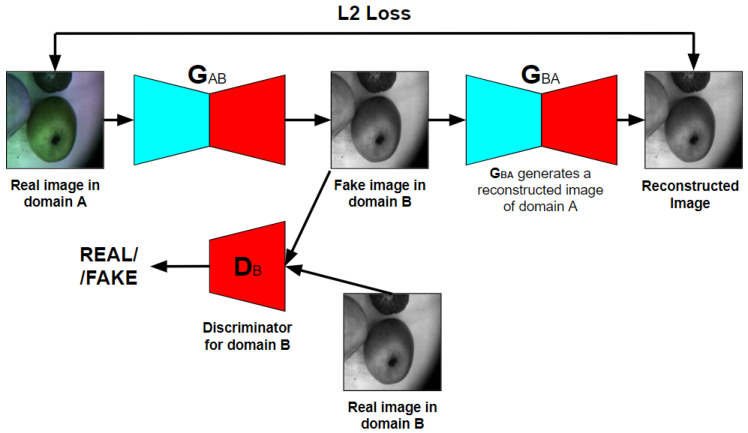
CycleGAN block diagram.

CycleGAN learns a translation mapping in the absence of aligned paired images. The image generated from *domain A* to *domain B* by the CNN-based generator ($G_{1}$) is converted back to *domain A* by another CNN-based generator ($G_{2}$), and vice versa, in the attempt to optimize the cycle-consistency loss in addition to the adversarial loss. The block diagram of CycleGAN is shown in Figure 3.

#### 3.1.3. Pix2PixHD

**Figure 4:**
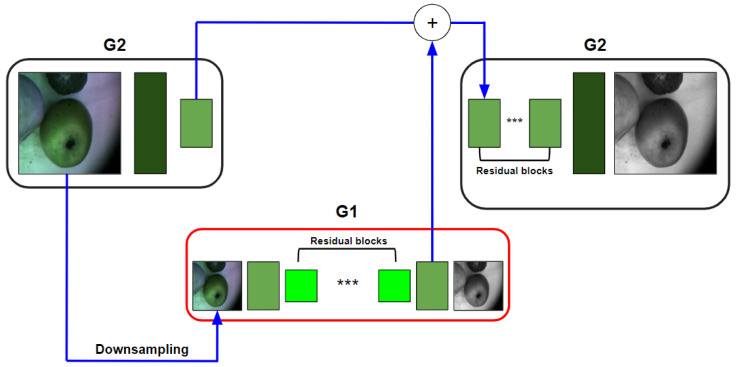
Pix2PixHD generator block diagram.

The Pix2PixHD model [36] is a modification of the solution realized in the Pix2Pix model, which includes several improvements including the Coarse-to-Fine generator, multi-scale discriminators, and improved adversarial loss. Pix2PixHD generally consists of global generator $G_{1}$ and local enhancer $G_{2}$ (see Figure 4, where *** are referred to the residual blocks). Throughout the training process, the global generator is initially trained, followed by the training of the local enhancer in a progressive manner based on their respective resolutions. Subsequently, all the networks are fine-tuned jointly. The purpose of this generator is to efficiently combine global and local information for the task of image synthesis. Three discriminators are used for effective detail capturing on multiple scales.

A significant performance boost was provided by the loss modification and two extra terms, $L_{FM}$-feature matching loss and perceptual loss, were added $L_{VGG}$ [93] as objective functions. The feature matching loss performs the stabilization of the training. It happens due the point that the generator has to produce natural statistics at multiple scales:

(5) $$L_{FM}\left(G,D_{k}\right)=\lambda_{FM}E_{y,x}\sum_{i=1}\frac{1}{N_{i}}\left[|\right|D_{k}^{\left(i\right)}\left(y,x\right)-D_{k}^{\left(i\right)}\left(y,G\left(y\right)\right){\left|\right|}_{1}]$$

where $D_{k}^{\left(i\right)}$ denotes the output of the *i*-th layer of the $D_{k}$ discriminator.

(6) $$L_{VGG}=\lambda_{VGG}E_{y,x}\sum_{i=1}\frac{1}{M_{i}}\left[|\right|F^{\left(i\right)}\left(x\right)-F^{\left(i\right)}{\left(G\left(y\right)\right)||}_{1}]$$

where $F^{\left(i\right)}$ denotes the *i*-th layer with $M_{i}$ elements of the VGG network.

#### 3.1.4. Mask R-CNN

**Figure 5:**
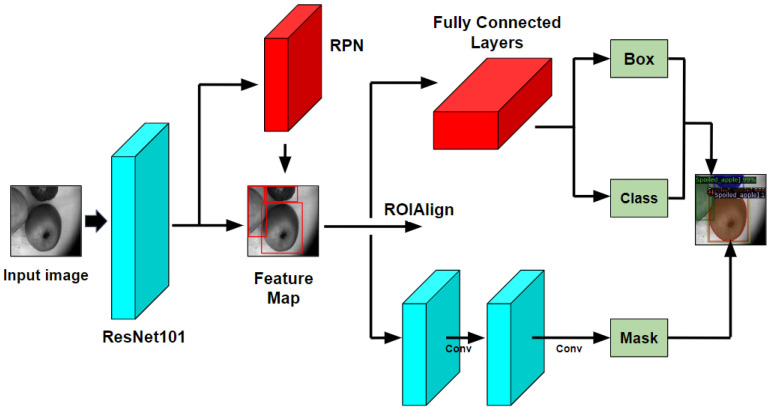
Mask R-CNN block diagram.

Mask R-CNN [94] is a CNN-based architecture that provides the instance segmentation of various objects in the images. These objects in images are usually called the Regions of Interest (ROIs). This is the latest version of the R-CNN model [95], where R-CNN stands for Regions detected with CNN. Firstly, R-CNN has been improved to Fast R-CNN [96], then to Faster R-CNN [97], and, finally, to Mask R-CNN. As it was mentioned earlier, in R-CNN based models the ROIs are detected with the CNN feature’s selective search. In Mask R-CNN, this selective search was improved to Mask R-CNN by adding the Region Proposal Network (RPN) in order to initiate and identify the ROIs and by adding a new branch for the prediction of the mask that covers the found region, i.e., an object in the image. The RPN and ResNet101 backbone allow for making the object detection (bounding boxes generation) and instance segmentation if there are several ROIs in one image and they have different sizes and partially overlap each other. Figure 5 presents a block diagram of Mask R-CNN architecture.

### 3.2. Performance Metrics

In this study, we compare the original VNIR images with the VNIR images generated by the Pix2PixHD model. To perform this, we considered the Mean Average Error (MAE), Mean Average Percentage Error (MAPE), Mean Squared Error (MSE), Root Mean Square Error (RMSE), Peak Signal to Noise Ratio (PSNR), Structural Similarity Index Measure (SSIM), and Feature Similarity Index Measure (FSIM) as follows:

(7) $$\mathrm{MAE}=\frac{1}{n}\sum_{i=1}^{n}\left|(y_{i}-x_{i})\right|$$

(8) $$\mathrm{MAPE}=\frac{100\%}{n}\sum_{i=1}^{n}\left|\frac{\left(y_{i}-x_{i}\right)}{y_{i}}\right|$$

(9) $$\mathrm{MSE}=\frac{1}{n}\sum_{i=1}^{n}{\left(y_{i}-x_{i}\right)}^{2}$$

(10) $$\mathrm{RMSE}=\sqrt{\frac{1}{n}\sum_{i=1}^{n}{\left(y_{i}-x_{i}\right)}^{2}}$$

(11) $$\mathrm{PSNR}=10\log_{10}\left(\frac{R^{2}}{\frac{1}{n}\sum_{i=1}^{n}{\left(y_{i}-x_{i}\right)}^{2}}\right)$$

(12) $$\mathrm{SSIM}=\left[{l(x_{i},y_{i})}^{\alpha }\cdot {c(x_{i},y_{i})}^{\beta }\cdot {s(x_{i},y_{i})}^{\gamma }\right]$$

(13) $$\mathrm{FSIM}=\left[{S_{PC}\left(x_{i},y_{i}\right)}^{\alpha }\cdot {S_{GM}\left(x_{i},y_{i}\right)}^{\beta }\right]$$

where $y_{i}$ is the generated or synthesized image, $x_{i}$ is the original image, *n* is the number of observations, *R* is the image maximum possible pixel value, *l* is the luminance, *c* is the contrast, *s* is the structure, α, β, and γ are the weights, $S_{PC}$ is the invariant to light variation in images, and $S_{GM}$ is the computation of image gradient.

We used precision, recall, mean Intersection over Union (IoU), mean Average Precision (mAP), and F1-score to verify the efficiency of the Mask R-CNN model on the synthesized VNIR pictures during the training and validation stages, which are defined as follows:

(14) $$\mathrm{Precision}=\frac{TP}{TP+FP}$$

(15) $$\mathrm{Recall}=\frac{TP}{TP+FN}$$

(16) $$\mathrm{IoU}=\frac{Area\;of\;Overlap}{Area\;of\;Union}$$

(17) $$AP=\sum_{n}\left({Recall}_{n}-{Recall}_{n-1}\right){Precision}_{n}$$

(18) $$F1-\mathrm{score}=2*\frac{Precision*Recall}{Precision+Recall}$$

Precision and recall are based on True Positives (TP), True Negatives (TN), False Positives (FP), and False Negatives (FN). TP denotes instances in which the model correctly predicts a specific object from a given class in images, TN denotes the instances in which the model correctly predicts an object that does not belong to a given class, and FP denotes the instances in which the model predicts a specific class, but the object does not actually belong to that class. In contrast, FN are the cases in which the model makes no prediction of a particular class, but the object actually belongs to one of the classes. The object classes are described in Section 3.4.

The *AP* is a region that lies beneath the precision–recall curve. The weighted mean of precisions at each IoU threshold, with the increase in recall from the preceding threshold as the weight, is how *AP* summarizes a precision–recall curve. It is calculated using (17), where ${Precision}_{n}$ and ${Recall}_{n}$ are the Precision and Recall at the *n*-th IoU threshold.

The mAP over all classes or overall IoU thresholds is calculated with the mAP score. AP is averaged over all the classes. There is no distinction between *AP* and mAP in this case. In our scenario, since AP is averaged across all the classes, there is no difference between AP and mAP. We calculated AP values for IoU = 0.50 (${AP}_{50}$), for IoU = 0.75 (${AP}_{75}$), for the objects with an area less than 32 squared pixels (${AP}_{S}$), for the objects with an area ranging from 32 to 96 squared pixels (${AP}_{M}$), and for the objects with an area higher 96 squared pixels (${AP}_{L}$).

### 3.3. Experimental Testbeds and Data Acquisition

In this section, we describe the apple fruits used for the experiments and present experimental testbeds for data collection:

(i) The experimental testbed for acquiring the dataset containing paired RGB and VNIR images of stored apples;

(ii) The experimental testbed for stored apple VNIR images collection containing VNIR images acquired by a multispectral camera.

The first testbed is designed for paired RGB and VNIR images collection in order to train and validate the GAN-based DL models for VNIR images translation from RGB images (see Section 3.3.1). The second testbed is used for the stored apples VNIR images collection as well as for the CNN-based model training and validation of postharvest decay zones detection and segmentation in the generated VNIR images (see Section 3.3.2).

#### 3.3.1. Experimental Testbed for Paired RGB and VNIR Imaging Data Collection

**Figure 6:**
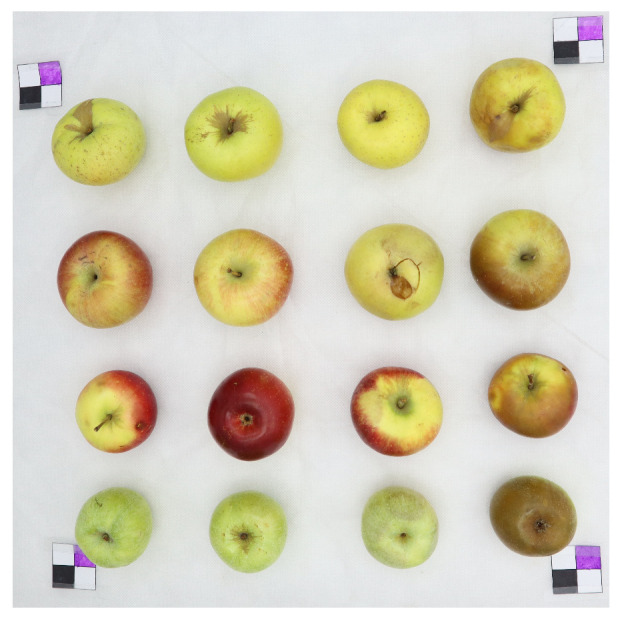
Apples selected for data collection.

We selected 16 apples of four kinds (“Delicious”, “Fuji”, “Gala”, “Reinette Simirenko”) and divided them into four rows according to their kind (each row corresponds to each apple kind). Each row contained four apples of different types, where every apple has different treatment from left to right: an apple with no treatment, a thoroughly washed and wiped apple, a mechanically damaged apple, and a shock-frozen apple supercooled under $-20^{\circ }$, respectively. The apple without treatment serves as a reference for each kind. A thoroughly washed apple indicates the removal of the natural protective wax layer from an apple. A mechanically damaged apple imitates the wrong storing conditions. A shock-frozen apple simulates the wrong storing conditions. Figure 6 shows these apples.

**Figure 7:**
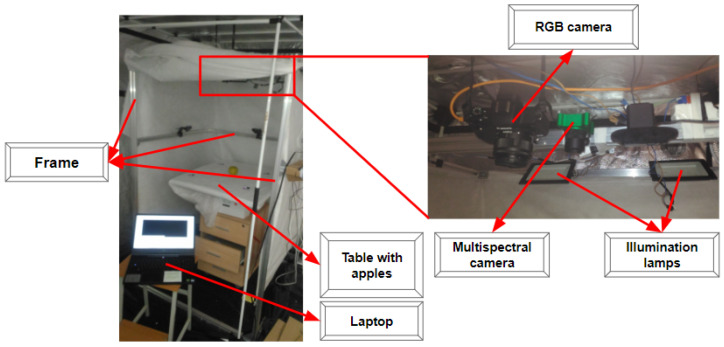
Experimental testbed for paired RGB and VNIR image capturing.

The first testbed is used for data collection under the recommended room storage conditions. The temperature ranges from 25 °C to 32 °C and Relative Humidity (RH) of 34% [98]. The testbed contains aluminum frames and is 1 m in length, 1 m wide, and 1.7 m high. Apples lie on a table with a white tray at the height of 1.3 m above the floor level. We also use SLR camera Canon M50 and the multispectral camera CMS-V1 CMS18100073 (CMS-V) attached at the middle top of the frame and connected to a PC laptop via the USB hub. The distance between the table with the apples on top and the camera is 500 mm. The lamps allowed us to simulate real storage conditions for apples as well as perform the collection of images under full and partial illumination. Detailed information about the acquired dataset and the first experimental testbed is described in [99]. Figure 7 shows the first testbed.

The multispectral camera CMS-V allows acquiring images in the range of 561–838 nm, including the visible and NIR ranges. This camera imager is characterized by the modified Bayer matrix made of a group of 3 × 3 pixels, called macro-pixel, filtering 3 × 3 (9) spectral bands. The raw image delivered by the camera is built of 9 interleaved spectral sub-images (8 colors + 1 Panchromatic) with the 1280 × 1024 pixels resolution. Each RGB image relates to 9 images from the following spectral bands *channel0* = 561 nm, *channel1* = 597 nm, *channel2* = 635 nm, *channel3* = 673 nm, *channel4* = 724 nm, *channel5* = 762 nm, *channel6* = 802 nm, *channel7* = 838 nm, and *channel8* (panchromatic channel) = 0 nm. The resolution of the nine sub-images is 426 × 339 pixels.

**Figure 8:**
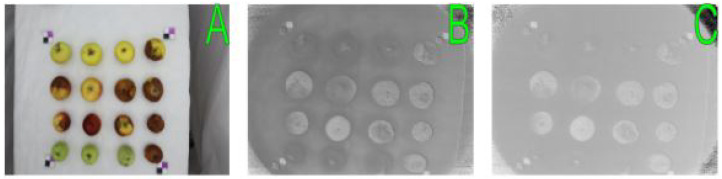
Types of images obtained during the experiments: (**A**)—RGB image of apples acquired under the full illumination; (**B**)—VNIR image of apples acquired under the full illumination (838 nm); (**C**)—VNIR image of apples acquired under the partial illumination (838 nm).

We acquired 1305 sequential RGB images and 1305 corresponding VNIR images in 838 nm range to see the decay dynamics in presented apples. The examples of images are shown in Figure 8.

#### 3.3.2. Experimental Testbed for VNIR Imaging Data Collection

**Figure 9:**
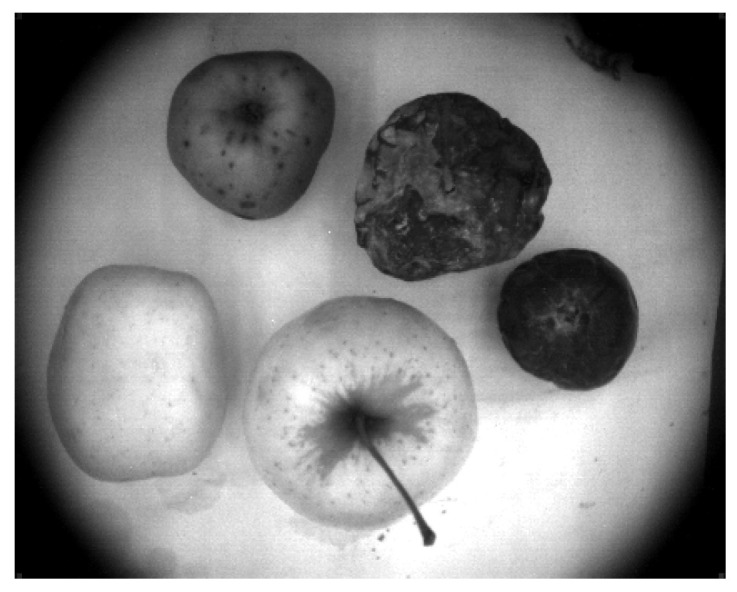
VNIR image of apples selected for data collection.

In this experiment, we selected 22 apples of the “Alesya”, “Fuji”, “Golden” and “Reinette Simirenko” seasonal types for data acquisition. The apples were between 8 and 10 cm in diameter, and most of them were multicolor with red and yellow sections. There were also some apples containing fungi zones, i.e., grey-brown moldy areas in apples, as the examples of apples stored under violated storage conditions. These apples were used in order to increase the data representation for early postharvest decay detection tasks in the stored apples using VNIR imaging data. These apples are demonstrated in Figure 9.

**Figure 10:**
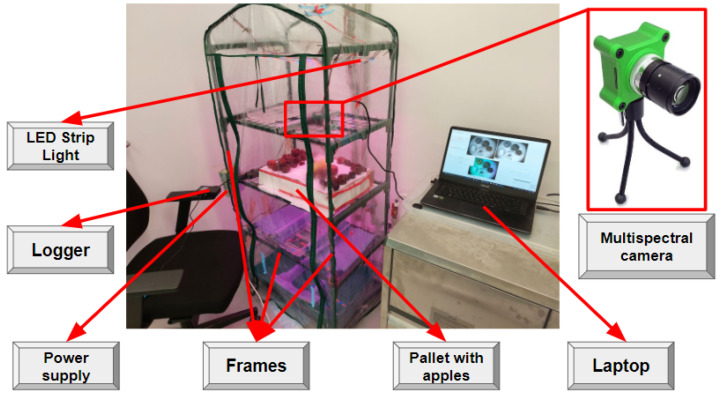
Experimental greenhouse for data acquisition.

The second testbed presented in Figure 10 is a greenhouse that includes silicon frames and five shelves, a plastic wrap, a multispectral camera, 10 LED strip lights with red/blue diodes, a power supply (total power is 150 Watt) for controlling the LEDs, a logger, and a pallet with apples. It can be used for the simulation of different processes related to plant breeding in various environmental conditions including extremely dry or wet modes. Temperature and humidity regulation in the testbed is provided with the LED strip lights, the plastic wrap, and several water pallets located on three lower bottom separate shelves.

The silica frames are the basic elements of a presented greenhouse characterized by the following dimensions 170 cm in height, 48 cm in length, and 67 cm in width. Two strip lights were fixed on each shelf while the multispectral camera and the pallet with the apples were fixed on the separate shelves (see Figure 10). Each selected strip has 60 LEDs with the wavelength of 650–660 nm (red light LEDs) and 455–465 nm (blue light LEDs) for highest chlorophyll concentration in plants to provide the most effective photosynthesis processes. This is also fair for crops and plants at the postharvest stages [100]. It is necessary to keep the quality of plant production which is another reason why these LED strip lights are used in the greenhouse. We rely on the power supply (12 V DC, 150 W, IP33) as the energy source for the SMD 5050 LED strip lights, and GL100-N/GL100-WL logger by Graphtech Corporation, supplied with the GS-TH sensor module, for temperature and humidity values registration during the data collection process.

For the VNIR image capturing, the multispectral camera CMS-V described in Section 3.3.1 was also chosen. The camera was connected via USB-A wire to the HP EliteBook 820 G3 Laptop with IntelCore i3-6100 CPU 2.30 GHz, where all the images were acquired and saved as JPG-files with 426 × 339 pixels.

**Figure 11:**
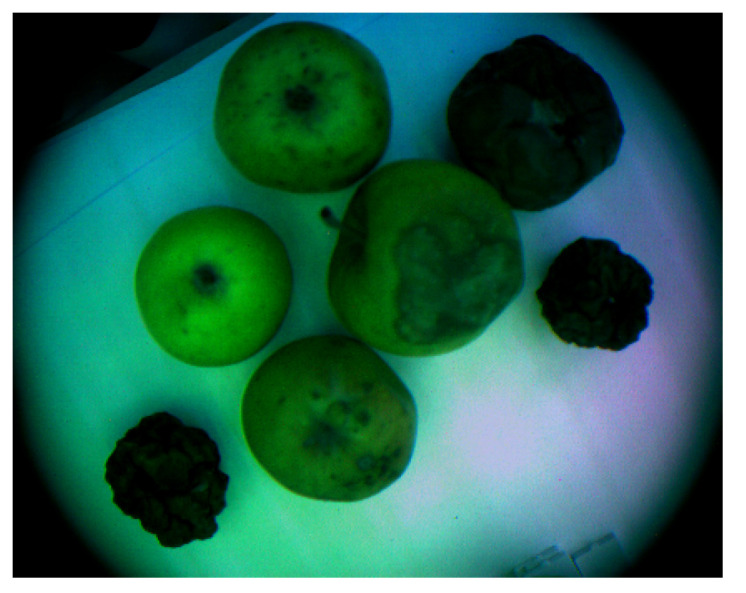
RGB image of apples selected for data collection.

We obtained 1029 sequential VNIR images in the 838 nm range collected from CMS-V camera’s *channel7*. These images were acquired under the temperature range from 35 °C to 40 °C and RH equal to 70% with the goal to simulate potential violation of the storage process of selected apples. This violation is necessary to speed up the decay processes in apples. We also collected 100 sequential RGB images (see the example in Figure 11) for the CNN-based model training and validation with the aim to demonstrate the up-to-date approach based on the combination of pre-trained GAN-based and CNN-based models. RGB sequential images had the dimensions of 339 pixels × 426 pixels × 3 channels (or simply 339 × 426 × 3).

### 3.4. Data Annotation

In order to apply a CNN-based deep learning model for the image instance segmentation, we used the Supervisely Ecosystem [101] for annotation and labeling of VNIR imaging data. It is worth reiterating here that we provide this labeling only for the VNIR images acquired with the testbed, described in Section 3.3.2 as these images were specially collected as the sequential VNIR imaging dataset for the DL model training and validation on early postharvest decay detection and segmentation of apples.

**Figure 12:**
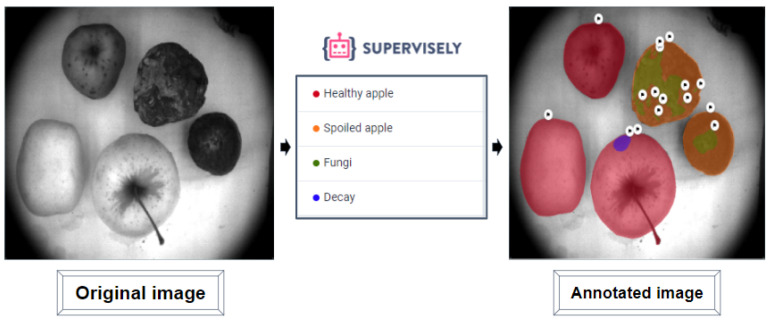
The example of image annotation and objects classes in Supervisely.

Four classes of objects in the images are defined as: *Healthy apple*, *Decay*, *Fungi*, and *Spoiled apple*. By the *Healthy apple* we understand the apples without any visible damages or spoiled zones in the images. The dark gray colored areas with the postharvest decay in apples were indicated as *Decay*. By *Fungi* we indicate white colored moldy zones in apples. Here we distinguish the postharvest decay zones marked as the *Decay* class, and moldy zones marked as the *Fungi* class. If an apple has objects of the *Fungi* class, it means that this apple is supposed to have been stored under the violated storage conditions, e.g., extreme temperature or humidity, which resulted in the apple’s full spoilage. The apples with only the postharvest decay zones (*Decay*) can be sent for recycling, while apples with moldy zones (*Fungi*) must be removed from others in order to prevent the spoilage of all samples. We also defined the *Spoiled apples* class: there are stored apples with more than 50 percent of spoiled areas (*Decay* objects) or moldy zones (*Fungi* objects) coverage. Figure 12 illustrates the procedure of image annotation.

## 4. Results and Discussion

### 4.1. Image-to-Image Models Comparison for VNIR Images Generation from RGB

In this section, we show the results of deep learning models based on generative adversarial networks comparison for VNIR images translation from RGB images. We provide this comparison on the dataset sequential RGB images and corresponding VNIR images in the 838 nm range presented in Section 3.3.1. To estimate the performance, we split the data into the train set (80%) and the validation set (20%). The augmentation techniques as Random Rotations, Shifts, Zoom, and Flips are implemented to increase the data representativity and to keep the model’s efficiency during the training and validation stages. We do not use the transformations such as Contrast/Brightness adjustments because they may lead to the information loss from the acquired VNIR imaging data. Taking into account that the image-to-image translation is also known as the translation from the *domain B* to *domain A* (or just *BtoA*), it was necessary to label *domain B* and *domain A* images from our acquired paired dataset. We identified the RGB images as *domain B* and *domain A* as the VNIR images. All models were evaluated by 200 epochs where the first 100 were implemented with the constant learning rate and the remaining 100 with linearly decreasing to zero. The models training and validation were realized via the Python scripts launched in Google Colab.

For the CycleGAN model, we use ResNet encoder–decoder architecture consisting of two downsampling layers, six ResNet bottleneck blocks and two upsampling layers. We also employ an Adam optimizer with the learning rate of 0.0002 and momentum parameters $\beta_{1}$ = 0.5 and $\beta_{2}$ = 0.999.

For the Pix2Pix model training, we fixed the same parameters: batch size = 1, $\beta_{1}$ = 0.5, $\beta_{2}$ = 0.999, and learning rate = 0.0002. The U-Net generator had 4 downsampling blocks. Optimization included the generator loss optimization step and the discriminator loss optimization step, respectively. Regularization parameters are as follows: $\lambda_{VGG}=\lambda_{Feat}=10$, $\lambda_{L_{1}}=100$.

For the Pix2PixHD model, we also implement the same parameters: Adam optimizer, batch size = 1, $\beta_{1}$ = 0.5, $\beta_{2}$ = 0.999, and learning rate = 0.0002.

**Figure 13:**
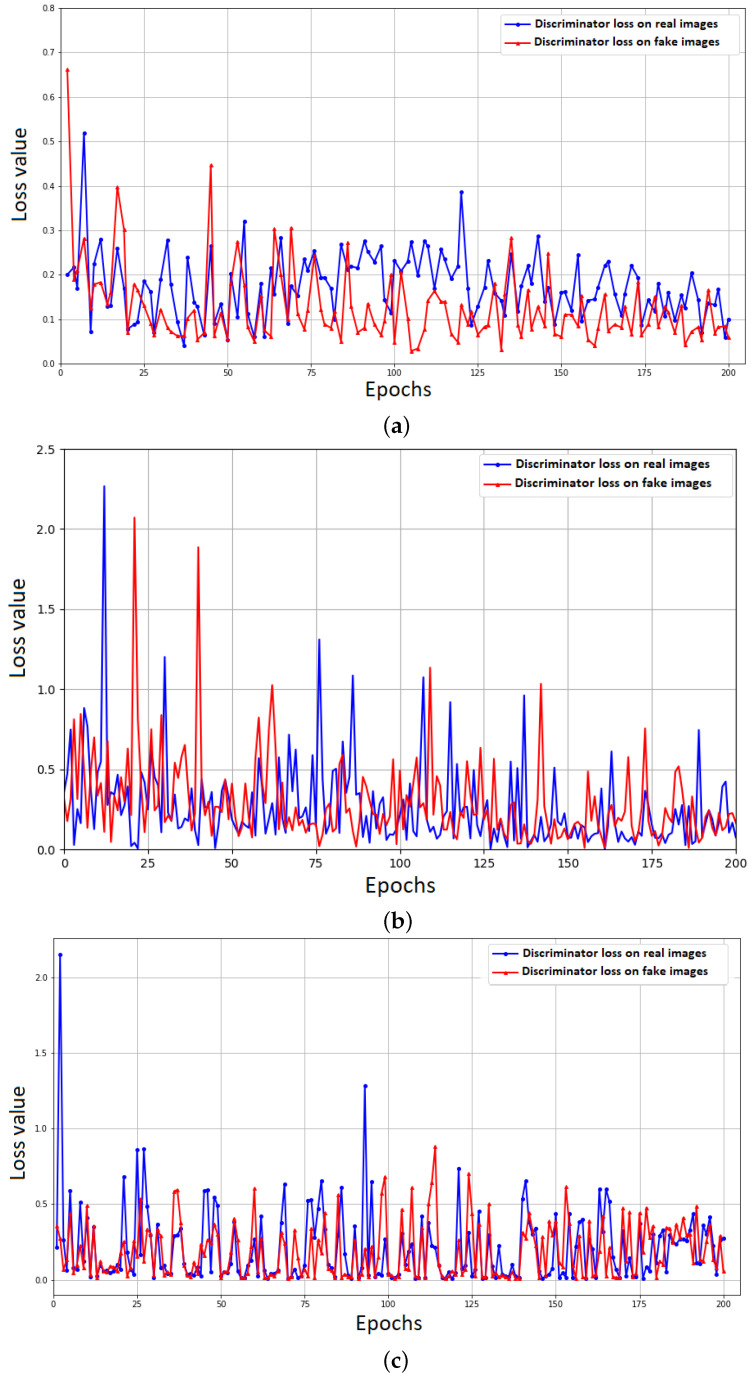
GAN-based models evaluation: (**a**) CycleGAN discriminator loss values during the training; (**b**) Pix2Pix discriminator loss values during the training; and (**c**) Pix2PixHD discriminator loss values during the training.

Figure 13 shows the discriminator values of CycleGAN (Figure 13a), Pix2Pix (Figure 13b), and Pix2PixHD (Figure 13c) models during the training stage. We show the model’s discriminator losses because they show the ability of GAN-based models to identify the quality of synthesized VNIR images by generator in comparison to original VNIR images.

**Figure 14:**
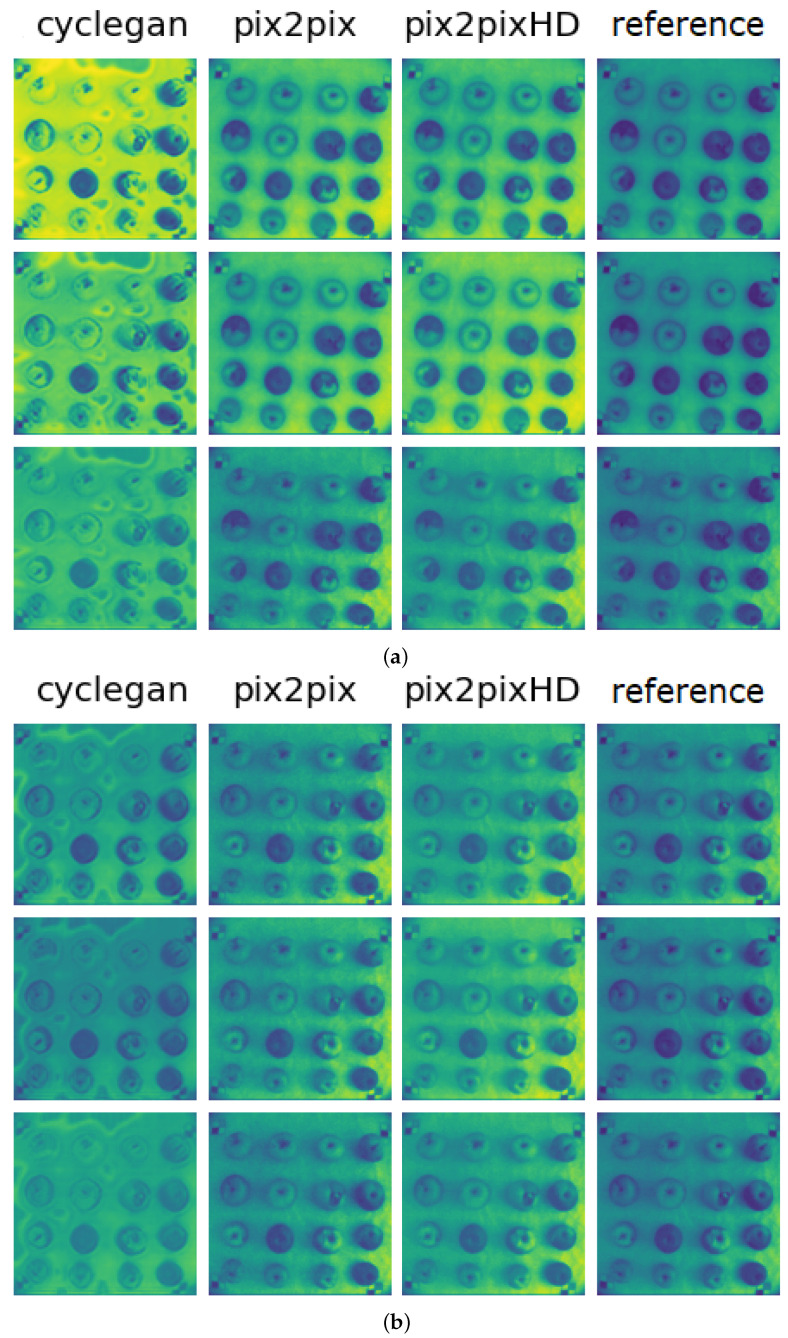
Examples of VNIR generated images in comparison to original VNIR image: (**a**) obtained under full illumination; and (**b**) obtained under partial illumination.

For selected GAN-based models we see that the training stage is unstable, but the discriminator losses tend to decrease over time. Pix2PixHD shows the lowest loss value in comparison to CycleGAN and Pix2Pix. For the models validation, we reconstructed the VNIR images using model weights acquired during the training. We used MAE, MAPE, MSE, PSNR and SSIM metrics to estimate the quality of VNIR reconstructed images in comparison with original VNIR images. Figure 14 shows these images (with ‘cyclegan’, ‘pix2pix’, ‘pix2pixHD’ labels, respectively) in comparison to the original VNIR image (‘reference’ label) via Python visualization tools.

Table 1 summarizes the results of considered models performance, where the results for Pix2PixHD model are highlighted with the black blod. Considering both the pixel-based and the image metrics, one can conclude on the promising results. The generated images look more or less similar to the original ones. The images containing apples, overall light intensity similar to the ground truth and the decay region are mainly preserved. However, all the models have particular artifacts. The CycleGAN model has the big stamp-like artifacts and there are a lot of missed decayed zones in the apples. In terms of metrics mentioned in Section 3.2, Pix2Pix and Pix2PixHD models perform the comparable and much better than others, and decay regions preserved relatively well, although the intensity level mismatch can be seen. Pix2PixHD models produce perceptually good images preserving importance for task features and the mean error level is equal to 0.6%. In terms of important metrics for the image quality estimation, such as PSNR and SSIM, the Pix2PixHD model showed higher values in comparison to Pix2Pix (46.859 against 46.433, and 0.972 against 0.955, respectively). Taking into account the results of this comparison, we decided to use the Pix2PixHD model for VNIR images generation from RGB during the next stages.

### 4.2. Segmentation of Generated VNIR Images for Early Postharvest Decay Detection in Apples

**Figure 15:**
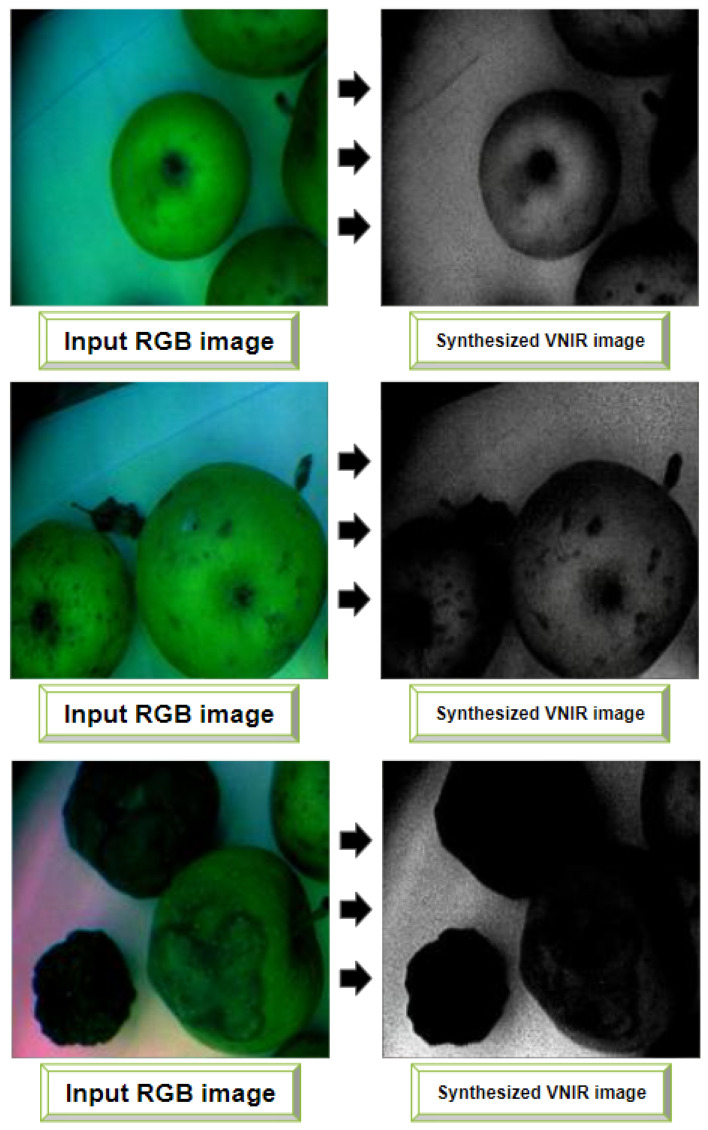
Examples of synthesized VNIR images with Pix2PixHD model weights.

In this section, we apply the CNN-based models for instance segmentation of generated VNIR images. Based on the results reported in Section 4.1, we use the Pix2PixHD model for the VNIR image generation. The dataset containing 456 images of stored apples (see Section 3.3.2) was used as the input for trained weights of the Pix2PixHD model to generate VNIR images. The examples of synthesized VNIR images from corresponding input RGB images are presented in Figure 15. Comparing the quality of new images with the images that were synthesizing during Pix2PixHD training stage (see Section 4.1), PSNR and SSIM values increased from 46.859 to 52.876 and from 0.972 to 0.994, respectively.

Mask R-CNN is used as the CNN-based model for the images instance segmentation. However, before applying Mask R-CNN to images, synthesized with Pix2PixHD, it was necessary to train Mask R-CNN on real VNIR images to detect and segment the fungi and decayed areas in stored apples. We used the labeled dataset containing 1029 VNIR images (see Section 3.3.2) for Mask R-CNN model training and validation. We report on the object classes used for data labeling in Section 3.4.

In this work, we implemented Mask R-CNN with the L1 as a loss function, ResNet50 as the backbone, Stochastic gradient descent (SGD) as an optimizer, and COCO weights to use Detectron2 library [102]. GaussianNoise, RandomGamma, RandomBrightness, and HorizontalFlip were applied as the data augmentation function to keep the efficiency of the proposed model during the training and validation stages. The model was developed in Python, and all calculations were realized in Google Colab.

In our experiment, we apply the cross-validation for Mask R-CNN model training on the dataset containing VNIR images. Cross-validation is a widespread technique helping avoid the overfitting during the model training on big data. In our case, we deal with the sequential images, i.e., one apple can be located in many images without any changes in position, which may resulted in improving the loss value after decreasing during the training procedure. During cross-validation, the data is usually split into several groups, called folds, where each group is used for the training and validation one by one. For example, if the dataset is separated into three folds, the pipeline is the following: (i) the first fold is a validation set, the second and third folds form the train set; (ii) the first and the third folds are train set, the second fold is a validation set; and (iii) the first and the second folds are training set, the third fold is a validation set. This pipeline is also fair for the cross-validation with four and higher folds distribution. By default, the number of folds, which is also called *k-folds*, is usually set equal to five or ten, but the *k-folds* may be different. In this work, we set the number of folds equal to two, three, six, and nine. We show the mean Average Precision values for each *k-fold* during Mask R-CNN models in Table 2.

**Figure 16:**
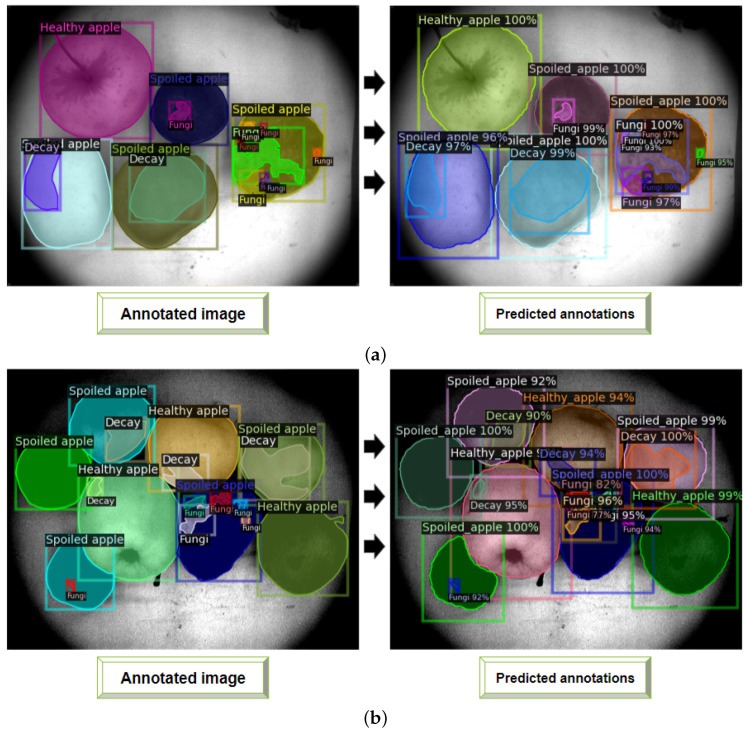
Comparison of object classes annotation in real VNIR images (**a**,**b**, on the left with ‘Annotated image’ label) to predicted object annotations (**a**,**b**, on the right with ‘Predicted annotations’ label) during Mask R-CNN model training.

The results for each object class segmentation (or per-category segmentation) during Mask R-CNN model during all folds are given in Table 3 and Table 4). We also used mAP and F1-score metrics to evaluate the segmentation quality during model training for folds distribution. Table 3 and Table 4 present the mean mAP and F1-score values for each fold, respectively. As can be seen, the number of folds leads to increasing of the metrics values and segmentation accuracy. This is a demonstration of a cross-validation technique in comparison to ordinary data splitting on the training and validation sets. Figure 16 shows the examples of VNIR images with predicted annotations of object classes (see Section 3.4) acquired during the Mask R-CNN model validation. Here we show the examples of synthesized and annotated images from *k-folds* = 9, as the distribution with the better mAP and F1-score values (see the column for *k-folds* = 9 with black bold in the Table 3 and Table 4). Even though the postharvest decay zones (*Decay* object class in Table 3 and Table 4) and the fungal areas (*Fungi* object class in Table 3 and Table 4) are detected with small values of an F1-score metric (58.861 and 40.968, respectively), a trained Mask R-CNN model allows for the detection and segmentation of spoiled apples (*Spoiled apple* object class), containing either decayed zones or fungal areas, or both, with an F1-score of 94.800, which is promising.

**Figure 17:**
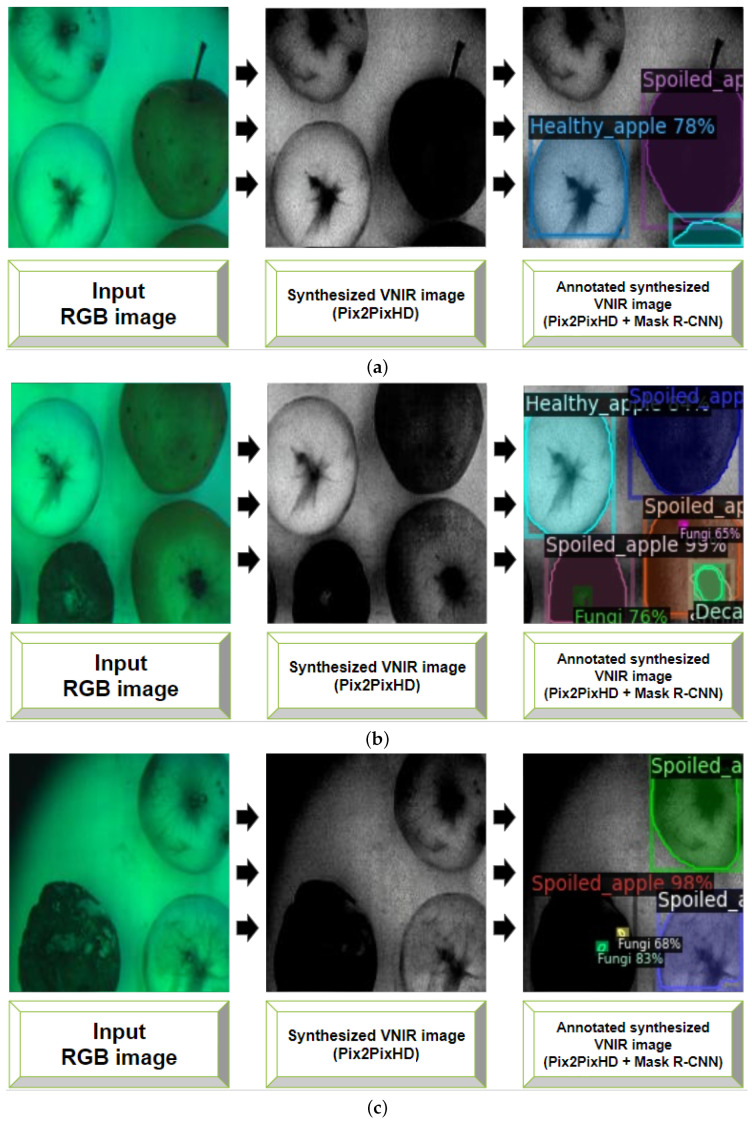
Synthesized VNIR images (**a**–**c**) segmentation with Mask R-CNN model.

Taking into account the results of Mask R-CNN evaluation on real VNIR imaging data and the results of the Pix2PixHD evaluation in comparison to other GAN-based models (see Section 4.1), we provide the proposed pipeline for segmentation of generated VNIR images. To estimate it we acquired the dataset containing only 456 sequential RGB images without the corresponded VNIR images (see Section 3.4). The images were acquired in the greenhouse (see Section 3.3.2) under the same environmental conditions (temperature range is from 35 °C to 40 °C, and RH is 70%, respectively). In order to simulate possible occasion during the real storage, spoiled apples with the decayed and fungi zones were added to healthy (non-damaged) apples. The concept is as follows: (i) we utilize a set of RGB images as input data; (ii) these RGB images are passed through a GAN-based model (specifically, Pix2PixHD with pre-trained weights in our case); (iii) VNIR images are generated from the input RGB images using Pix2PixHD; and (iv) the generated VNIR images are fed into a CNN-based model (specifically, Mask R-CNN with pre-trained weights) to obtain these images with predicted annotation masks. Figure 17 shows the examples of images which were synthesized and segmented with the proposed pipeline. As it can be seen in Figure 17b,c, the proposed approach helps detect and segment the decayed zones separately from the fungi zones in the stored apples. All computations were also provided in Google Colab.

### 4.3. Early Postharvest Decay Detection in Stored Apples Using Generated VNIR Imaging Data on an Embedded System

To evaluate the applicability of a GAN- and CNN-based models in real-life scenarios we conduct an experiment using the NVIDIA Jetson Nano embedded system [103]. The goal of the experiment is to validate the model’s ability to handle video streams with varying frames per second (FPS).

**Figure 18:**
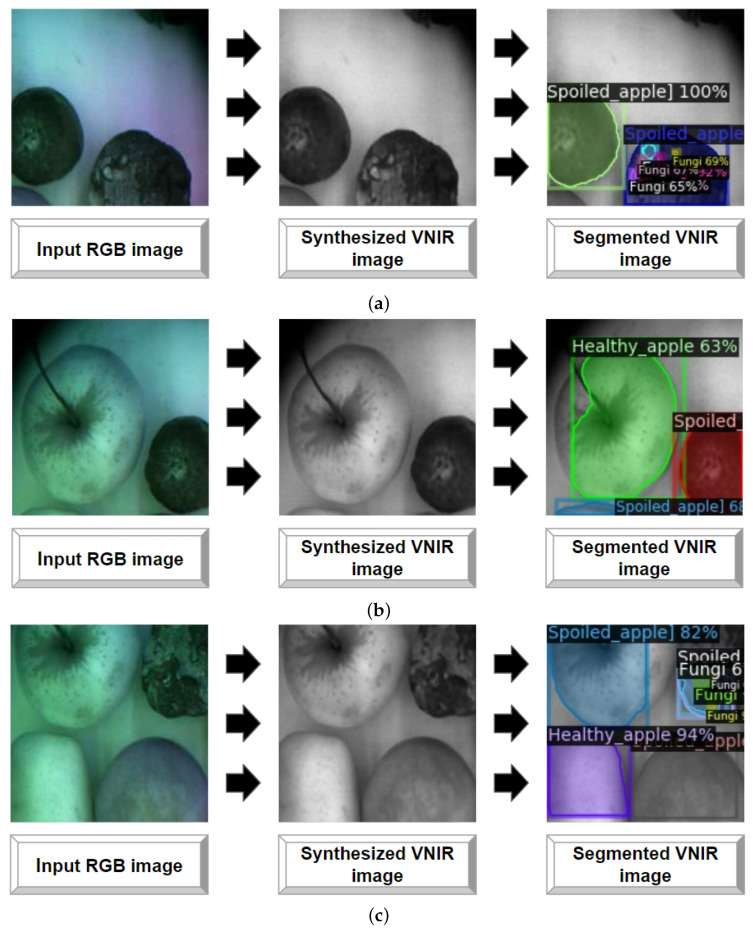
Generated and segmented VNIR images (**a**–**c**) using Jetson Nano.

We used 100 RGB images. Input RGB images are characterized by the size of 256 pixels. A GAN model was used to generate VNIR images from input images and processed over 100 images at an average rate of 17 FPS. The generated images were then tested with Mask R-CNN, resulting in an average rate of 0.420 FPS. Low FPS in Mask R-CNN can be attributed to its complexity compared to Pix2PixHD. As the two-stage detection model that performs instance segmentation by detecting objects and generating pixel-level masks for each object, it requires more computational resources. Figure 18 shows the examples of VNIR images generated and segmented using the NVIDIA Jetson Nano based on the input RGB data.

### 4.4. Discussion

In this section, we compare our results with other relevant research works in the field of application of NIR imaging data and deep learning techniques for early postharvest decay and fungal zones prediction in stored apples. The proposed approach is based on the joint application of GAN and CNN techniques for artificial generation and subsequent segmentation of VNIR images. However, in order to segment the decayed and fungal zones in artificially generated VNIR images, we had to train and validate a CNN technique on the real VNIR images containing these zones in stored apples. To perform this, we acquired the dataset of VNIR images (see Section 3.3) and then trained and validated the Mask R-CNN model (see Section 4.2).

Taking into the account the ability of Mask R-CNN to provide the multi-class instance and semantic segmentation (see Section 3.1.4), we trained the model not only to detect and identify the quality of apple (*Healthy apple* or *Spoiled apple*, see Section 3.4), but also to detect and predict the decayed and fungal zones separately from each other. Novelty is that the model is trained and validated to identify the quality of stored apples by taking into account the presence of decayed and fungal areas in the apples themselves. In this context, an apple is classified as *Spoiled apple* if it contains the decayed or fungal zones, whether they are separate or combined. Conversely, if an apple does not exhibit any decayed or fungal zones prior to storage stage, i.e., during the VNIR image collection, it is classified as a *Healthy apple*. However, if the decayed and/or fungal zones emerge in the apple during the storage stage, its classification transitions from a *Healthy apple* to *Spoiled apple*.

Relevant works in this area can be classified into three main groups according to main tasks: (i) defective apples detection based on the internal quality parameters [104,105]; (ii) early defect detection in apples [104,106]; and (iii) early fungi detection in apples [73,107,108]. Table 5 presents a comparative study of these works.

The authors applied various tools and methods based on machine learning for detecting the defected and diseased zones in wide NIR ranges (400–2350 nm, globally) with detailed spectral information on the diseased zones. The most relevant and similar approach to the current research is reported in [104], where a YOLO v4 model in sorting machine for real-time detection of defects in “Red Fuji”, “Golden Delicious”, and “Granny Smith” apples is implemented. The authors used the RGB and corresponded NIR images in the range of 850 nm of the apples in the machine’s sorting line. Moreover, the ability of trained YOLO v4 models to detect with bounding box ‘calyx’ and ‘stem’ zones separately from ‘defect’ zones was demonstrated. In this work, we applied the Mask R-CNN not only to detect (with bounding box) and segment (with mask) the decayed and the fungal areas in stored apples, but also to identify the quality of apples as diseased (*Spoiled apple*) if such zones are detected by the model. F1-score and mAP values for *Decay* and *Fungi* zones are not that high. These problems can be fixed in our future work by obtaining more VNIR images containing the fungal and the decayed areas in order to increase the data representation during the model validation. On the other hand, the results for *Spoiled apple* (apple contains *Fungi* and/or *Decay* zones) segmentation are 98.350 and 98.375, respectively, which is promising. Finally, the proposed approach is for an apple quality control during the storage stage, i.e., before sending the stored apples to the fruit sorting machine. The system, which could generate VNIR images without a multispectral or hyperspectral camera based only on the input RGB images with segmented fungal and decayed zones, if they occur in stored apples, can be applied as an additional stage for the fruit and vegetable control before sending them to a sorting machine.

In [106], the authors compared several Faster R-CNN, YOLO v3-Tiny, and YOLO 5s models for early decay (or bruise) detection in apples. The approach proposed in this work showed promising results in terms of the mAP metric (98.350 for Mask R-CNN validation, in our case, against 96.900 for Faster R-CNN, 99.100 for YOLO v3-Tiny, and 96.600 for YOLO 5s), and the selected model was trained to segment the decayed and fungal zones in apples, while authors in [106] trained the models to identify and predict the apples without (‘No bruise’), with a small (‘Mild bruise’) and significant (‘Severe bruise’) decayed areas in apples. The authors also acquired the NIR images in spectral range of 900–2350 nm, while in this work the images from 838 nm range were used in order to make sure that the diseased zones in VNIR images are visible in the RGB images as well.

In [105], the authors trained and validated U-Net and the improved U-Net model for the defect segmentation in VNIR images of apples. In this work, we have demonstrated the semantic segmentation of decayed and fungal areas with an advanced experimental methodology. We simulated ordinary and extreme storage conditions during the paired RGB and VNIR images collection procedures. Taking this into account, we achieved a relevant value for the diseased apples segmentation in terms of the F1-score metric.

We have demonstrated the potential for the postharvest decay and fungi prediction for stored apples. However, it can be scaled to other crops that are widely used in food production, e.g., carrots, tomatoes, cucumber, fruits or bananas. For example, the system that allows the generation and segmentation of VNIR images can be applied for segmentation and prediction of such fungi as *Sclerotinia sclerotiorum* or *Botrytis cinerea*. ’Sclerotinia’ and ’Botrytis’ fungal zones have similar morphology and, if they occur in plants, it is a nontrivial task to identify one fungi variety from another one using only RGB imagery or visual estimation of the internal fungal traits with human eyes [109]. The system supplied with the trained and validated DL technique based on the GAN and CNN models can assist the user with the additional spectral information about each fungi acquired from the generated VNIR images. It is useful for more precise antifungal activities during the food quality control.

Another potential scenario is the application of the proposed research for the preharvest diseases and the defect detection for the plants both growing in natural environments and in artificially controlled systems. For example, it can be a robot moving platform or unmanned aerial vehicle without a hyperspectral camera, but with an embedded system that may generate and segment the NIR imaging data from the input RGB one. However, DL technique should be trained, tested and validated precisely, as the proposed system has to detect and segment not only the diseased plants from the healthy ones, but also to detect the kind of defect (damage, decay, fungi variety) with the following suggestion of spoiled fruit processing.

## 5. Conclusions

NIR imagery provides detailed information about the diseased areas in stored fruits, which is why the hyperspectral cameras containing thousands of bands are used for food quality monitoring at postharvest stages. However, hyperspectral devices are expensive and are not friendly for the farmers and sellers’ usage. In this article, we have presented the approach based on the GAN and CNN DL techniques for early postharvest decay zones and fungi areas detection and prediction in stored apples using synthesized and segmented VNIR images.

The conclusions of this work are as follows:

- The analysis of Pix2Pix, CycleGAN, and Pix2PixHD models, which are widely used GAN techniques, and their application to a dataset containing paired 1305 sequential RGB images and 1305 sequential VNIR images of stored apples of different varieties and various pre-treatments. The images were acquired under the full and partial illumination with the goal to simulate real storage conditions.
- Comparison of the real VNIR images with the VNIR images synthesized by selected GAN based models. The VNIR images generated via Pix2PixHD a 0.972 score for the SSIM metric.
- The training and test of Mask R-CNN on another dataset containing only 1029 sequential VNIR images of apples under violated storage conditions. Within this test, an F1-score of 58.861 is achieved for the postharvest decay zones and F1-score 40.968 for the fungal zones detection. The spoiled apples with the decayed and fungal zones are detected and segmented with F1-score 94.800.
- Testing of the proposed solution on an embedded system with AI capabilities. We used 100 RGB images of stored apples as an input data for NVIDIA Jetson Nano, and the time processing of VNIR images generation by Pix2PixHD showed 17 FPS. The detection and segmentation by Mask R-CNN achieved 0.42 FPS.

The proposed approach is a promising solution able to substitute expensive hyperspectral imaging devices for early postharvest decay prediction tasks in postharvest food quality control.

## Figures and Tables

**Table 1:** Image-to-image models comparison for RGB to VNIR images generation.

Models	MAE	MAPE	MSE	PSNR	SSIM
CycleGAN	0.067	0.105	0.01127	27.375	0.856
Pix2Pix	0.004	0.006	0.00003	46.433	0.955
Pix2PixHD	**0.004**	**0.006**	**0.00003**	**46.859**	**0.972**

**Table 2:** Comparison of Average Precision for Mask R-CNN model.

k-Folds	mAP	mAP${}_{50}$	mAP${}_{75}$	mAP${}_{S}$	mAP${}_{M}$	mAP${}_{L}$
2	64.251	90.205	65.606	37.202	75.980	97.412
3	67.652	90.354	65.348	35.400	75.290	96.290
6	67.026	90.950	67.055	38.188	74.609	98.871
9	67.993	91.120	64.871	31.575	75.181	97.257

**Table 3:** Results on per-category segmentation by Mask R-CNN using mAP metric.

Category	mAP			
	k-Folds=2	k-Folds=3	k-Folds=6	k-Folds=9
*Healthy apple*	94.785	95.154	93.951	**98.350**
*Spoiled apple*	87.839	92.567	93.678	**93.997**
*Decay*	53.509	53.408	54.620	**57.562**
*Fungi*	31.581	30.609	34.285	**39.967**

**Table 4:** Results on per-category segmentation by Mask R-CNN using F1-score metric.

Category	F1-Score			
	k-Folds=2	k-Folds=3	k-Folds=6	k-Folds=9
*Healthy apple*	95.640	95.589	94.799	**98.375**
*Spoiled apple*	88.120	93.134	94.689	**94.800**
*Decay*	53.309	53.213	54.850	**58.861**
*Fungi*	31.686	37.247	35.126	**40.968**

**Table 5:** Comparative table of relevant research works.

References	Task	NIR Images Range, nm	Technique	Metric	Value
[104]	Real-time apple defect inspection	850	YOLO v4	F1	92.000
[105]	Apples surface defect segmentation	460–842	U-Net	F1-score	87.000
[105]	Apples surface defect segmentation	460–842	the improved U-Net	F1-score	91.000
[106]	Early bruise detection in apples	900–2350	Faster R-CNN	mAP	96.900
[106]	Early bruise detection in apples	900–2350	YOLO v3-Tiny	mAP	99.100
[106]	Early bruise detection in apples	900–2350	YOLO 5s	mAP	99.600
[107]	Moldy core detection in apples	400–850	CARS-PLS-DA model	Accuracy	87.880
[73]	Codling Moth detection in apples	900–1700	Gradient tree boosting	F1-score	97.000
[108]	Moldy core detection in apples	200–1100	BP-ANN	Accuracy	95.000

## Data Availability

Not applicable.

## Abbreviations

The following abbreviations are used in this manuscript:

NIR	Near Infrared Image
VNIR	Visible Near Infrared Image
AI	Artificial Intelligence
CV	Computer Vision
ML	Machine Learning
SVM	Support Vector Machines
RF	Random Forest
kNN	K-Nearest Neighbors Algorithm
GTB	Gradient Tree Boosting
DL	Deep Learning
CNN	Convolutional Neural Network
GAN	Generative Adversarial Network
ROI	Regions of Interests
SBC	Single Board Computer
RH	Relative Humidity
